# Biodegradation of Natural Rubber: Microcosm Study

**DOI:** 10.1007/s11270-021-05171-7

**Published:** 2021-05-22

**Authors:** Francesca Bosco, Chiara Mollea

**Affiliations:** Department of Applied Science and Technology, DISAT, Politecnico Di Torino, C.so Duca degli Abruzzi 24, 10129 Torino, Italy

**Keywords:** Natural rubber, Biodegradation, Filamentous fungi, Respirometric analysis, Microcosm

## Abstract

In the present work, natural rubber (NR) biodegradation, by means of a microbial consortium, naturally selected in a tyre dump soil, has been evaluated. To this purpose, prepared soil microcosms were incubated for 236 days, at room temperature, and natural light/dark cycles. The effect of primary C-source and fresh soil addition, soil aeration, and humidity maintenance has been monitored by means of microbiological and respirometric analysis, dry weight loss determinations, and SEM micrographs. During the incubation, in biodegradation microcosms (BD), containing NR samples, the produced CO_2_ was significantly higher than that of biotic controls (BC). Furthermore, after 236 days, a NR dry weight loss of 15.6%, in BD microcosms, was registered, about four-fold higher than that registered in BC control (3.7%). Obtained results confirmed that the naturally selected microbial consortium was able to use NR as the only C-source and to biodegrade it. The positive effect of soil mixing evidenced that the biodegradation process was mainly carried out by aerobic biomass, especially filamentous fungi, as confirmed by microbial counts and SEM observations. Results obtained in the microcosm study provided useful information in terms of soil aeration and nutrient amendment in view of a future biodegradation process scale-up.

## Introduction

Natural rubber (NR) is used to manufacture products essential for the everyday life; consequently, the worldwide demand for rubber grows continuously and it almost entirely gravitates towards the NR production because it is currently unfeasible to synthetically reproduce NR, in particular due to its branched chemical structure (Sriring et al., 2018). NR is mainly extracted from the *Hevea brasiliensis* Müll. Arg. tree, typical of the tropical Amazonian forests. The latex, collected from the tree by tapping the trunk, mainly contains *cis*-1,4-polyisoprene units, 87% dry weight, while the remaining 13% is represented by proteins, lipids, carbohydrates, and minerals (Bottier, 2020). The produced quantities reach numerous millions of tons per year: NR has unique properties (i.e. high tensile strength, elasticity, and flexibility) which make it a raw material of great interest (Zhao et al., 2019).

Rubber products belong to assorted application areas, such as the manufacturing, medicine, household, and transport ones. Among all these applications, rubber is greatly used in the medicine field for the production of disposable medical or personal protective equipment (PPE) (Mente et al., 2016). In particular, in the year 2020, the use of PPE has grown exponentially for the protection from the novel coronavirus (SARS-CoV-2) infection: 65 billion gloves have been consumed monthly worldwide, of which 0.5 billion only in Italy (Prata et al., 2020). These rubber amounts, related to the PPE, are just a portion of those consumed yearly worldwide: in the year 2019, about 14 million tons of NR have been used (Wang et al., 2020). The increased demand of PPE, related to the SARS-CoV-2 virus circulation, is currently due to an amplified usage by the medical personnel but also by common people in their everyday life (Nowakowski et al., 2020). This has led to a significative advance in the volume of disposed PPE, including a huge amount of not-reusable rubber latex gloves. In particular, in order to limit the virus diffusion, common people wear these PPE for limited time periods and change them very frequently. The related amount of rubber wastes, difficult to be managed, will represent a risk for the public health, because these wastes can be a vector for the SARS-CoV-2 virus, but also a risk for the ecosystems, due to the uncontrolled and direct discard in the environment (Di Maria et al., 2020; Klemeš et al., 2020).

Different strategies have been proposed or applied through the years to overcome the damages related to rubber waste disposal; they can be treated on-site or exported, burned, or dumped, stockpiled, and discarded in landfills. Unfortunately, often these operations are conducted illegally causing additional pollution or other negative consequences (Winternitz et al., 2019); for example, rubber wastes buried in agricultural soils can cause soil infertility with high economic repercussions for the plantations. In the year 2020, as regard the SARS-CoV-2 virus diffusion is concerned, the blending of the lightweight latex gloves with other wastes has been proposed or their incineration has been planned to recover energy (Nowakowski et al., 2020). This last solution is not environmental friendly because it often creates air pollution problems; furthermore, the recovery value of the waste rubber process is only a low one (Adhikari et al., 2000).

The NR structure can be weakened by abiotic degradation processes: mechanical, photo-, thermal, and chemical degradation (Chen & Qian, 2003; Ibrahim et al., 2020; Sadaka et al., 2012). As a matter of fact, all of these processes are often based on the use of hazardous chemical solvents, large amounts of energy, and can also led to the generation of unhealthy micro-plastics.

An effective alternative, to overcome all the rubber waste disposal problems, can be represented by the biodegradation which allows to avoid or limit the environmental pollution–related complications (Andler, 2020). Natural rubber biodegradation depends on various factors and, among them, the polymer characteristics (i.e. molecular weight, crystallinity, the presence of functional groups, substituents, or additives), the type of involved microorganisms, and the possible polymer pre-treatments play a major role (Ali Shah et al., 2013). The biodegradation process evolves through four consecutives transitions, which are the bio-deterioration, the bio-fragmentation, the assimilation, and the mineralization. During the first one, the chemical and physical properties of the polymer are altered, while during the second step, the enzymatic cleavage allows the polymer breakdown. The assimilation is the uptake of the molecules by the microorganisms; finally, the mineralization is the conclusive phase, characterised by the release of CO_2_ and H_2_O, in aerobic conditions, and of CO_2_, CH_4_, and H_2_O, in anaerobic ones (Pathak & Navneet, 2017). In particular, rubber, being a natural product, undergoes various bio-mineralization cycles also when it is in the cross-linked form, as it is the case of latex gloves or vulcanised rubber (Ali Shah et al., 2013).

Numerous bacteria and fungi, able to degrade NR as a carbon and energy source, are present in natural environments, and among them, those of the soil microflora have been frequently isolated and studied to examine their rubber biodegradation potentiality (Imai et al., 2011).

Bacterial rubber degradation has been extensively studied since 1936, when, for the first time, the isolation of rubber-degrading bacteria was described by means of latex overlay plates, where the bacterial colonies grown forming translucent halos (Jendrossek et al., 1997). Both Gram( +) and Gram( −) bacteria are able to biodegrade this polymer; bacteria belonging to the group of the CNM (*Corynebacterium*, *Nocardia*, and *Mycobacterium*) and the actinomycetes, such as *Actinoplanes*, *Streptomyces*, and *Micromonospora*, have been enumerated by Yikmis and Steinbüchel (2012) as promising biodegrades. In recent years, also the bacteria belonging to *Gordonia* spp. have been considered for their rubber biodegradation ability together with that of biofilm formation capability (Braga et al., 2019; Huong, 2020).

The first knowledge about the NR biodegradation capability of filamentous fungi was reported by De Vries in the year 1928, who described the NR biodegradation, in liquid culture, by *Aspergillus* sp. and *Penicillium* sp.: the obtained NR weight decrease reached 15.5% after 19 months of incubation. In the following years, numerous studies confirmed the possibility to biodegrade NR by means of these fungal species and new ones were added (Rose & Steinbüchel, 2005). Recently, Pathak and Navneet (2017) enumerated, among the species involved in natural and synthetic rubber polymer degradation, *A. niger*, *Aspergillus flavus*, *Fusarium solani*, *Pycnoporus cinnabarinus*, and *Mucor rouxii* as the most prevalent ones. Studied fungi, for the most part, were directly isolated from the rubber surface or from the soil, while the degradation was carried out in liquid culture, or on the rubber surface directly, or inoculating the strains in the soil together with buried NR fragments (Mollea & Bosco, 2020). In recent years, examples of NR biodegradation studies by means of pure fungal cultures with the soil burial method have been reported. *Cladosporium fulvum*, isolated from soil, was able to degrade wide NR discs, buried in the soil, allowing to obtain a 5.3% rubber weight loss after 60 days of incubation (Nawong et al., 2018).

When a biodegradation approach is taken into consideration, it is necessary to initially stimulate the activity of those microorganisms capable of remove toxic compounds possibly present in the rubber composition, that is, mainly fungi; for example, Stevenson et al. (2008) studied the ability of the fungus *Resinicium bicolor* to remove toxic components. Subsequently, the biodegradation can be carried on by other degrading microbial species.

Most of the studies regarding rubber microbial degradation have been conducted, at a lab scale, in liquid cultures often utilizing a minimal mineral medium enriched with the rubber as the only carbon source. As reported by Yahya et al. (2011), the microbial degradation rate of rubber is influenced by the rubber formulation but also by the type of the involved microorganisms, and their interactions with the environment. The biodegradation rates of NR are slower than those of other natural polymers; this is particularly due to the branched chemical structure of the rubber itself (Qu et al., 2016; Rose & Steinbüchel, 2005). For all these reasons, it can be useful to conduct lab-scale studies by setting-up soil microcosms exploiting the natural soil microflora; this is a good approach to evaluate, also for prolonged incubation periods, the microbial capability to degrade rubber, the potential activity of the indigenous soil biomass, and also the most effective bioremediation strategy. The biodegradation of rubber is a slow process; therefore, it requires long incubation periods (i.e. from some weeks to months) to allow the biomass growth and significative modifications on the rubber mass/structure (Ali Shah et al., 2013). A recent example is that of a full-scale soil burial test which has proven the high biodegradability of NR in the presence of the natural soil microflora; a mass loss of 16.2% after only 45 days and of 38.3% at the end of the incubation time (90 days) were obtained burying thin probes in a rich nutrient soil and exposing them to the soil microflora (Mastalygina et al., 2020). Tests carried out in microcosms, at the lab scale, do not always guarantee reproducible results on-site, due to the influence of chemical, physical, and biological factors, but they allow to take these aspects into account, simulating them in controlled conditions, and to verify the rubber biodegradability (Bosco & Mollea, 2019; Bosso et al., 2015). This last aspect can be of interest considering, for example, the influence of the natural weathering on rubber weakening. When rubber is exposed to direct or indirect environmental conditions (e.g. light, heat, oxygen, and moisture), the unsaturated backbone is modified. In particular, oxygen can easily attack the rubber, degrade the polymer, and can cause rubber hardening or softening (Muniandy et al., 2017). At the same time, the environmental conditions directly influence the microbial classes and the degradative pathways involved in the biodegradation process; for example, in the presence of available O_2_, aerobic microorganisms predominate in the polymeric material degradation and grow producing new microbial mass together with CO_2_ and H_2_O as final products (Ali Shah et al., 2013).

The aim of the present work was the evaluation of NR biodegradation by means of a microbial consortium naturally selected in a soil collected in a dump of tyres. The main parameters (i.e. primary C-source and fresh soil addition, soil aeration, and humidity maintenance) that control the biodegradation process have been monitored by means of microbiological and respirometric analysis; furthermore, the dry weight loss has been determined and the microscopic modifications of NR have been evaluated by means of scanning electron microscope (SEM) micrographs.

## Materials and Methods

The experimental tests on the natural rubber (NR) biodegradation has been carried at laboratory scale, in microcosms containing soil derived from a dump of tyres and NR sheets.

### Natural Rubber

The natural rubber (NR) incubated in the microcosms is commercially known as Standard Malaysian Rubber Grade L (SMRL); it consists of *cis*-1,4-polyisoprene and is characterised by a molecular weight of about 500 kDa and an unsaturation level of 98% (mol/mol). NR samples were cut in round shape (Ø 2.54 cm) from discs prepared with a diameter of about 15 cm and a thickness of 0.2 cm.

### Soil Preparation and Characterisation

The soil used for the biodegradation experiment was collected from an illegal dump of tyres, located in a rural area, in the Piemonte region, in north-western Italy. It was sampled to a 20-cm depth, passed through a 2-mm sieve and stored at + 4 °C, in glass containers, until its utilization.

#### Soil–Water Content

The soil humidity (H), expressed as its water content, was determined by weighing small portions (5–10 g) and drying them, at 105 °C, at least for 24 h and until the weight reached a constant value. The humidity % was calculated as indicated in Eq. 1 (soil quantities are expressed in grams):

1 $$H\%=\left[\left(\mathrm{wet soil}-{\mathrm{soil dry weight}}_{105^\circ\mathrm{{C} }}\right)/\mathrm{wet soil}\right]\times 100$$

#### Water Holding Capacity (WHC)

Twenty-five grams of soil were saturated with 100 mL of distilled water and left at room temperature for 2 h. The bottom of a Buchner funnel was then sealed with a moistened filter paper: the soil sample was spread uniformly on the filter and the system was attached to a vacuum pump in order to withdraw water from the sample. Finally, the soil sample was recovered and dried at 105 °C until a constant weight was reached.

The WHC% was calculated as indicated in Eq. 2:

2 $$WHC\%=\left[\left({\mathrm{soil}}_{\mathrm{saturated}}-{\mathrm{soil} \mathrm{dry weight}}_{105^\circ{C} }\right)/{\mathrm{soil dry weight}}_{105^\circ{\rm C} }\right]\times 100$$

In Eq. 2, “soil_saturated_” is the mass of the initial soil–water mixture, while “soil dry weight _105 °C_” is that of the dried soil; quantities are expressed in grams.

#### Soil pH Determination

The pH of the soil was determined with the potentiometric method using a pH meter. The measures have been conducted by wetting the soil (10 g), contained in a beaker, with 25 ml of distilled water, or 1 M KCl solution, or 0.01 M CaCl_2_ solution.

Samples were maintained for 2 h under continuous stirring. Then, each suspension was left to settle for 10 min and the pH of the liquid phase was directly measured inside the beaker by means of a pH meter. The reported soil pH value is the average of the three ones obtained with distilled water and the two salt solutions.

#### Organic Carbon Content of the Soil

A portion of soil, 5 g, was dried at 105 °C until a constant value of the weight was reached. After that, the dried soil was left for 4 h at 650 °C and then weighted. The organic carbon content % (C_organic_%) was determined as indicated in Eq. 3:

3 $${\mathrm{C}}_{\mathrm{organic}}\%=\left[\left({\mathrm{soil dry weight}}_{105^\circ\mathrm{{C} }}-{\mathrm{soil final weight}}_{650^\circ{\rm C} }\right)/{\mathrm{soil dry weight}}_{105^\circ{\rm C} }\right]\times 100$$

#### Soil Microbiological Characterisation

The soil has been microbiologically characterised in order to detect the number of autochthonous microorganisms and to discriminate between bacteria and fungi. For this purpose, the Malt Extract Agar (MEA, malt extract 20 g/l, glucose 20 g/l, peptone 2 g/l, agar 20 g/l) was prepared adjusting the pH value at 4 or 5, by adding an HCl solution; the medium has been steam sterilised (121 °C, 2 atm, 20 min) and poured in the Petri dishes at a temperature of about 50 °C. The pH 4 is favourable for fungal growth, while pH 5 allows the bacterial one. The microbial characterisation has been realised for the soil prior the biodegradation experiment at different incubation time. Two different methods have been applied: the “soil dilution” (by spreading and inclusion of inoculum) and the “soil plate” ones.

Soil dilution has been prepared by mixing soil and sterile distilled water in a proportion of 1:2 (weight:volume). The suspension has been maintained in agitation, at 125 rpm, with glass balls, in sterile conditions for 1 h. At the end, the suspension has been water diluted (1:100, 1:1000, 1:10,000) and 100 µl of each dilution has been homogeneously spread over the surface of the MEA plates for the spreading method. In the case of the inclusion method, 500 µl of each dilution was poured in the middle of the Petri dish, covered by liquid MEA, then left to solidify. Dishes were incubated in the dark, at room temperature for 48 h. In both the cases, at the end of the incubation, microbial colonies have been counted and the obtained results expressed as CFU/g_soil_.

As regard the soil plate method is concerned, soil samples (50 or 100 mg) have been placed in the middle of the Petri dishes. After that, 500 µl of sterile distilled water was added and the mixture was well mixed to obtain a homogeneous suspension. Finally, 20 ml of liquid MEA, prepared at pH 4 or pH 5, was poured on the soil suspension. Also, in this case, the prepared Petri dishes were maintained in the dark and at room temperature; the microbial count was performed after 48 h of incubation.

### Microcosm Set-up

The set-up of the soil microcosms was carried out into sealed glass jars (capacity 1 l), having a rubber seal integrated in the stopper. An amount of 200 g of soil was poured and dispersed at the bottom of each jar; moreover, distilled water was added to bring the soil humidity to the 35% of the measured WHC. When necessary, each microcosm component has been steam sterilised at 121 °C and 2 atm pressure, for 20 min. Furthermore, during the time course of the biodegradation, Czapek medium (CZ medium, composition: Glucose 30 g/l, NaNO_3_ 3 g/l, K_2_HPO_4_ 1 g/l, MgSO_4_ 0.5 g/l, KCl 0.5 g/l, FeSO_4_ 0.01 g/l), with or without glucose, was added.

**Fig. 1:**
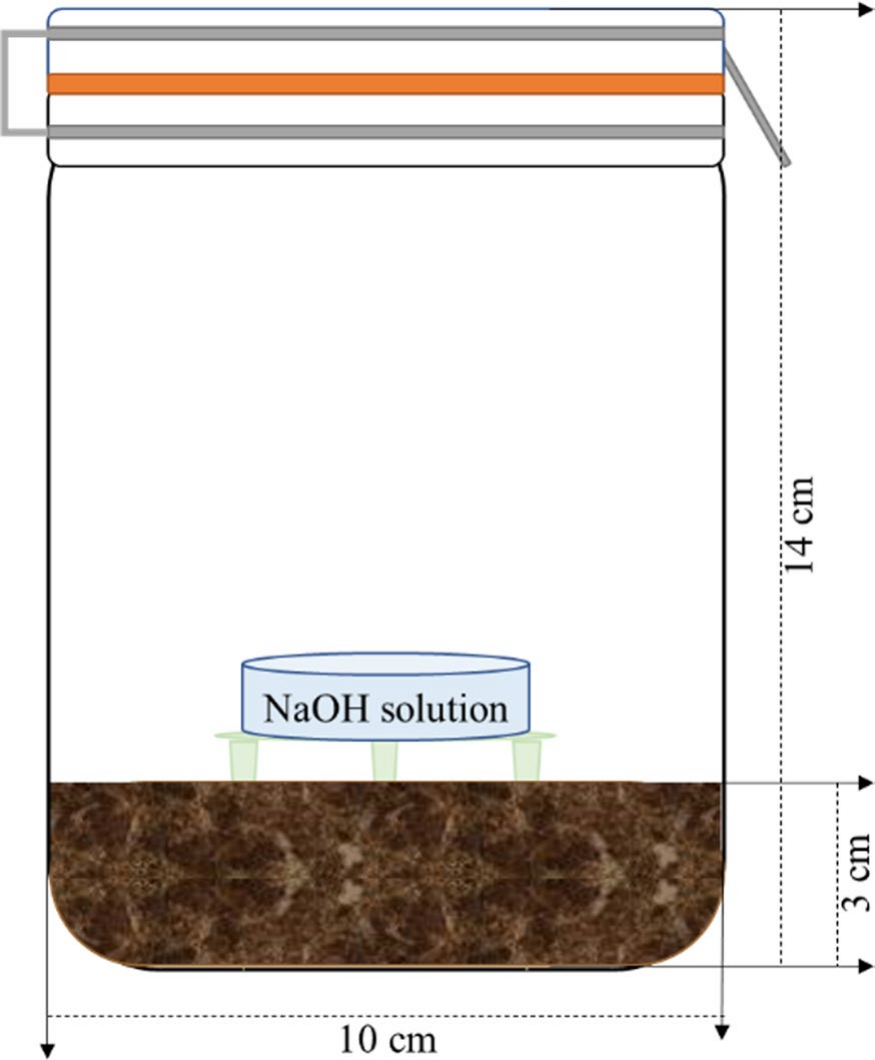
Soil microcosm set-up in a glass jar

The apparatus for CO_2_ capture was positioned on top of the soil in the middle of each microcosm; as it is shown in Fig. 1, it was composed by a plastic base with three pins (Ø 8 cm, height 3 cm) to support the container for the NaOH solution (capacity 100 ml), used for CO_2_ capture. Once the CO_2_ capture apparatus was positioned, the microcosms were carefully sealed and maintained at room temperature and at the natural light/dark cycle.

The microcosm set-up is listed in Table 1; for each line, microcosms were prepared in triplicate. As regard the microcosms containing the NR samples is concerned, two of the four samples were placed at the top of the soil, while the others were buried at 1 cm depth.

**Table 1:** Microcosm line set-up

	Number of NR samples	NR sample steam sterilisation	Soil steam sterilisation
Abiotic control (AC) line	4	Yes	Yes
Biotic control (BC) line	0	No	No
Biodegradation (BD) line	4	No	No

#### Respirometric Determinations

The evaluation of the respirometry activity, inside the sealed microcosms, was performed by means of the measure of the produced CO_2_, captured by a NaOH solution (1.5 N), using an acid–base titration with HCl 1.5 N, BaCl_2_ 1 M as precipitating agent, and a phenolphthalein solution, as a colour indicator (Mollea et al., 2005).

### Analyses of the NR Samples

NR samples were periodically sampled from the microcosms and analysed to evaluate the degree of the biodegradation process. The extent of the biodegradation was quantified by means of dry weight loss determination while changes in the rubber morphology were examined with SEM analyses.

#### NR Washing Method

The NR sheets, sampled from the microcosms, were washed to remove soil particles and the adhered biomass following the procedure previously described by Mollea and Bosco (2020).

#### NR Dry Weight

The dry weight of the rubber fragments was evaluated at the time of the microcosms set-up and, periodically, during the incubation in the microcosms. NR fragments, washed or not, have been dried in an oven at 55 °C for 24 h and then weighted by means of an analytical balance.

#### SEM Analyses

The morphology of the NR surface was investigated by means of SEM analyses. NR, washed or not, was fixed overnight with a 5% glutaraldehyde solution and then analysed using a LEO/Zeiss 1450VP SEM (beam voltage: 5 kV). The NR pieces were pinned onto conductive adhesive tapes and then coated with gold prior to be analysed. The imaging used mode was the secondary electron (SE mode) one.

## Results and Discussion

### Soil Characterisation

#### Chemical-Physical Characterisation

The soil used for the biodegradation experiments, collected from an illegal dump of tyres in north-western Italy, was classified as silty loam and characterised by a WHC of 66% and by a humidity of 10% (w/w). It resulted weakly alkaline, pH 7.5, and its organic carbon content was equal to 11%.

#### Microbial Characterisation

The soil was microbiologically characterised by means of plate counts of the culturable microorganisms with two different methods, soil dilution, by spreading or inclusion, and soil plates. The CFU of bacteria and filamentous fungi were determined on Malt Extract Agar (MEA) plates at two different pH values, 5 and 4, respectively. Three replicates were set-up for all the analyses, and the obtained mean values are reported.

The characterisation was performed, at the microcosm set-up (t_0_), for the non-incubated soil. Furthermore, in order to identify the quantitative and qualitative changes in the soil indigenous microflora after 97 days of incubation, the characterisation was repeated, in the presence or not of NR samples, for BD and BC microcosms respectively.

In Table 2, the CFU/g_soil_, obtained with the soil dilution and soil plate methods, are reported. As regard the soil dilution method is concerned, values obtained for the spreading and inclusion techniques at pH 4 were different (5.1 × 10^5^ and 1.2 × 10^4^ respectively), about one order of magnitude, whereas at pH 5, the difference was negligible. This could be explained by the fact that the inoculation method mainly influences the growth of filamentous fungi, generally aerobic; on the contrary, the bacterial one is less affected. Therefore, the tested soil shows a high number of indigenous microorganisms, both bacteria (3.9 × 10^6^ CFU/g_soil_) and fungi (5.1 × 10^5^ CFU/ g_soil_). The CFU/g_soil_ values obtained with the soil plate method at pH 4 were about doubled with respect to those at pH 5 for the same order of magnitude (1.8 × 10^3^ at pH 4 and 1.0 × 10^3^ at pH 5). At the same time, these results were lower than those found with the soil dilution method, independently of the pH value.

**Table 2:** Viable cell counts: results (CFU/gsoil) obtained for the non-incubated soil (t_*0*_) with the soil dilution and soil plate methods at pH 4 and pH 5

	CFU/g_soil_	
	pH 4	pH 5
Soil dilution		
Spreading	5.1 × 10^5^ ± 2.3 × 10^5^	3.9 × 10^6^ ± 1.6 × 10^6^
Inclusion	1.2 × 10^4^ ± 1.0 × 10^4^	1.6 × 10^6^ ± 0.5 × 10^6^
Soil plates	1.8 × 10^3^ ± 1.1 × 10^3^	1.0 × 10^3^ ± 0.6 × 10^3^

**Fig. 2:**
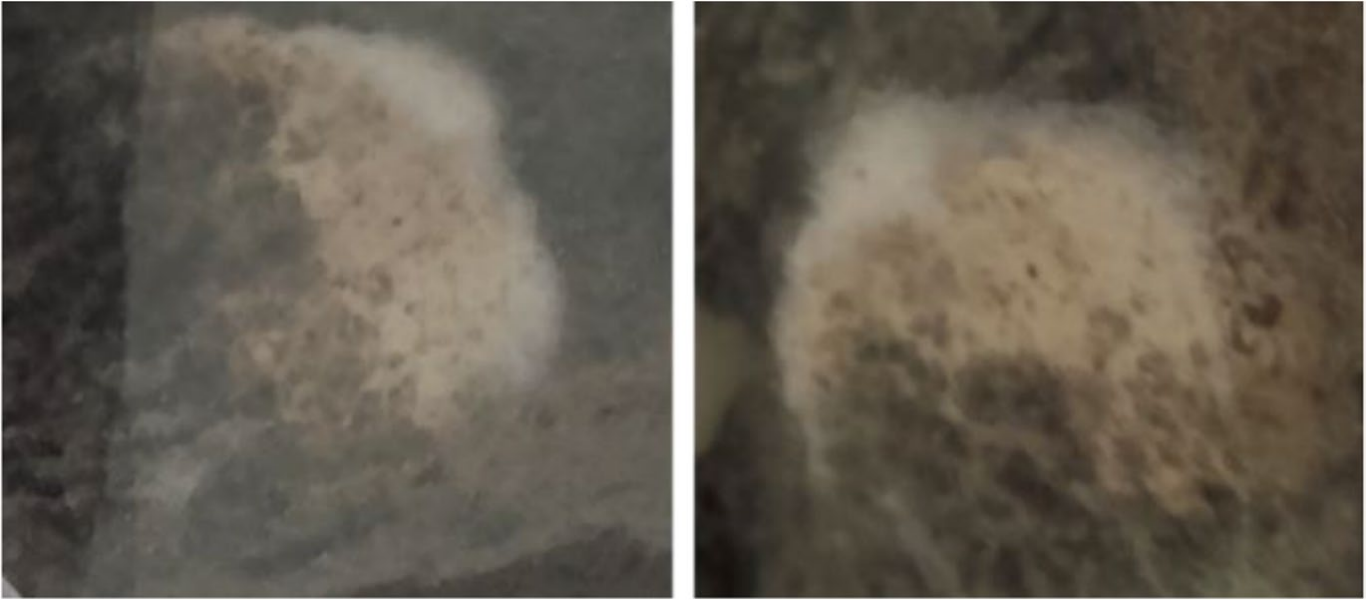
Details of the NR sheets placed on the surface of the soil: fungal mycelium covering the surface at the 97*th* day of incubation

The same methods, used for the microbiological characterisation of the non-incubated soil, were applied to the soil, added or not with NR sheets (BD and BC lines, respectively), incubated in the microcosms for 97 days (Fig. 2). Soil samples were taken from each of the three microcosms of the BC and BD lines; the results obtained with the two soil suspension inoculation methods, spreading and inclusion, have been averaged and are shown in Table 3. Therefore, for each line, a mean value (CFU/g_soil_) for every “microcosm type-pH value” combination was determined.

**Table 3:** Viable cell counts for the soil in the BC and BD microcosms: results (CFU/gsoil) obtained after 97 days of incubation at pH 4 and pH 5 are shown. Ratios of the CFU/gsoil values between the BD and BC lines and between the BD lines, at the two pH values, are also reported

	CFU/g_soil_	
	pH 4	pH 5
BC line	9.4 × 10^4^ ± 10 × 10^4^	8.0 × 10^5^ ± 6.9 × 10^5^
BD line	3.8 × 10^5^ ± 3.9 × 10^5^	1.2 × 10^6^ ± 4.6 × 10^5^
	CFU/g_soil_ /CFU/g_soil_	
BD line/BC line	1.9	3.2
BD line_pH5_/BD line_pH4_	3.0	

The results referred to the 97th day of incubation clearly show that in the presence of NR sheets (BD line), the CFU/g_soil_ number for filamentous fungi and bacteria was quite higher (3.8 × 10^5^ and 1.2 × 10^6^ at pH 4 and pH 5 respectively) than that obtained in the BC microcosms at both the pH values (9.4 × 10^4^ and 8.0 × 10^5^ at pH 4 and pH 5 respectively).

The CFU/g_soil_ results were compared with each other by means of the “BD/BC lines” ratio, calculated at the same pH value, and the “BD line_pH5_/BD line_pH4_” ratio, as shown in Table 3. The values of the BD/BC line ratio indicate that in the BD microcosms, both bacterial and fungal colonies were more numerous than those present in the BC controls: more precisely, about two-fold (1.9) at pH 4 and three-fold (3.2) at pH 5. This behaviour could be attributed to the presence of NR, in the BD microcosms, as an additional carbon source. Moreover, comparing the results obtained in the BD lines at the two pH values (BD line_pH5_/BD line_pH4_ ratio), the ratio value of 3 simply indicates that the number of bacterial colonies was higher than the fungal ones and not necessarily that the role of bacteria was predominant in the NR biodegradation.

Another interesting comparison, reported in Table 4, was the number of microorganisms in non-incubated (t_0_) and incubated soil at the 97th day. In the BC line, it was possible to observe a drastic decrease in both bacterial (− 64%) and fungal colonies (− 61%) compared to those initially present in the soil not incubated. On the contrary, in the BD line, the bacterial colonies remained almost stable (− 16%) while there was a noticeable increase in the fungal ones (+ 47%). At the same time, after 97 days of incubation, in the BD microcosms, it has been observed that the NR sheets, positioned on the surface of the soil, were extensively colonised by abundant fungal hyphae: NR can represent an alternative C-source for these microorganisms able to secrete extracellular enzymes, as it has already been demonstrated in liquid cultures by Mollea and Bosco (2020).

**Table 4:** CFU/gsoil values of the non-incubated soil and after 97 days of incubation, for the soil in the BC and BD lines, at pH 4 and pH 5, showed results are an average of those obtained with the two different soil inoculum dilution method. The variation % in the microbial colonies is also showed

pH	CFU/g_soil_ non-incubated soil (t_0_)		CFU/g_soil_ 97 days of incubation	Variation %
4	2.6 × 10^5^	BC line	9.4 × 10^4^	− 64
		BD line	3.8 × 10^5^	+ 47
5	2.8 × 10^6^	BC line	8.0 × 10^5^	− 61
		BD line	1.2 × 10^6^	− 16

**Fig. 3:**
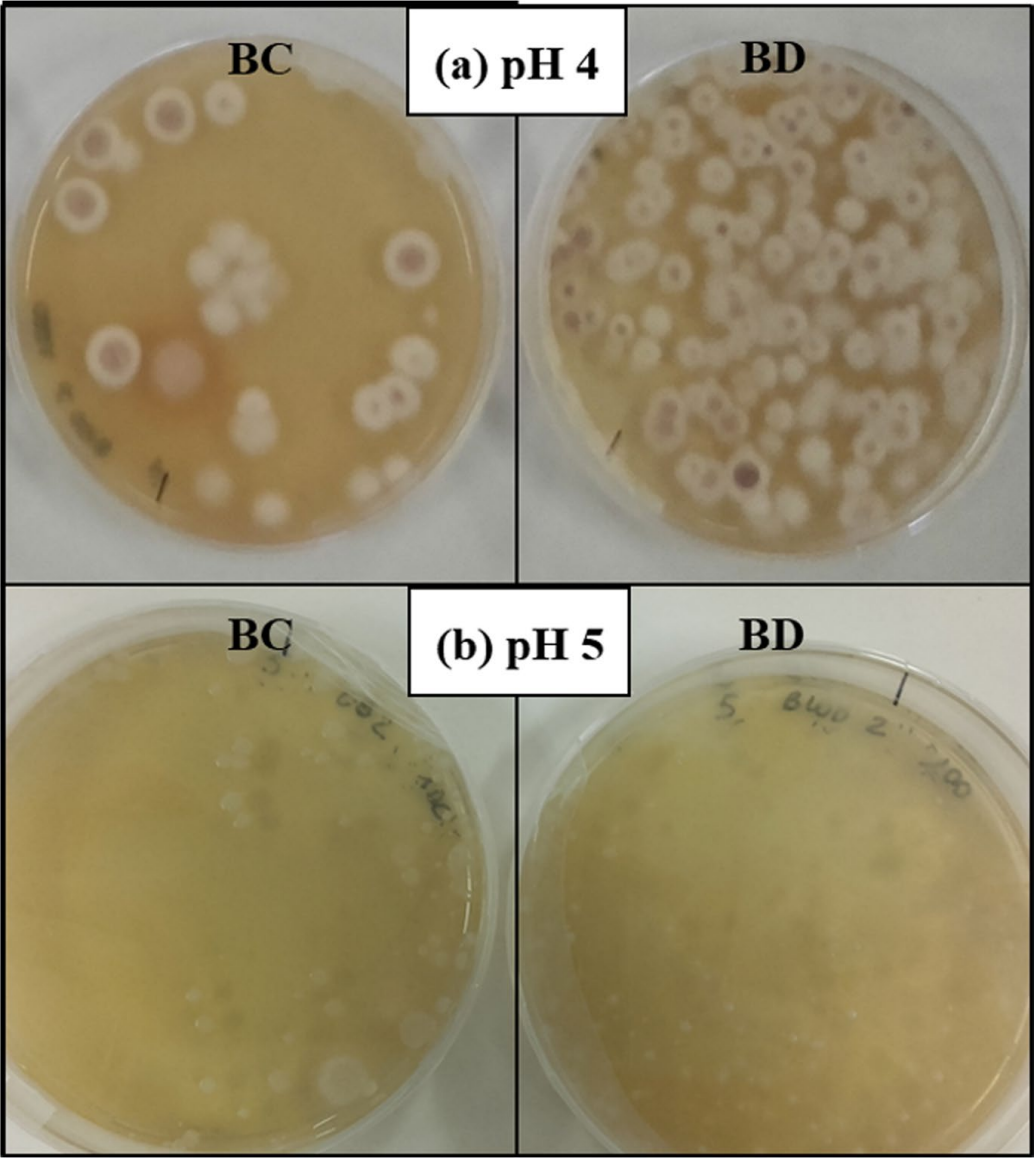
Viable counts on MEA at pH 4 (***a***) and pH 5 (***b***) for the soil sampled from the BC and BD microcosms at the 97*th* day *of incubation (1:100 dilution)*

The results reported in Table 4 agreed with the images in Fig. 3, at 97 days of incubation, where a comparison of the microorganisms in BC and BD lines is shown. In the plates of MEA prepared at pH 4, the difference in the number of fungal colonies between the BC line (left) and the BD control (right), at 97 days of incubation, was noteworthy (Fig. 3a). On the contrary, in plates of MEA at pH 5, a significant difference in the number of bacterial colonies was not evident (Fig. 3b).

### Stimulation of the Microbial Activity in Microcosm

The biomass activity and growth have been evaluated, in all the microcosms, by means of respirometry measurements; more precisely, CO_2_ production was monitored.

The relative humidity (RH%) of the soil was maintained at 35% by adding distilled water in the time course of the incubation. The presence of available water in the microcosm soil is fundamental because, as reported by Mastalygina et al. (2020), water can be absorbed in the NR structure and makes it susceptible to the activity of soil-degrading microorganisms.

**Fig. 4:**
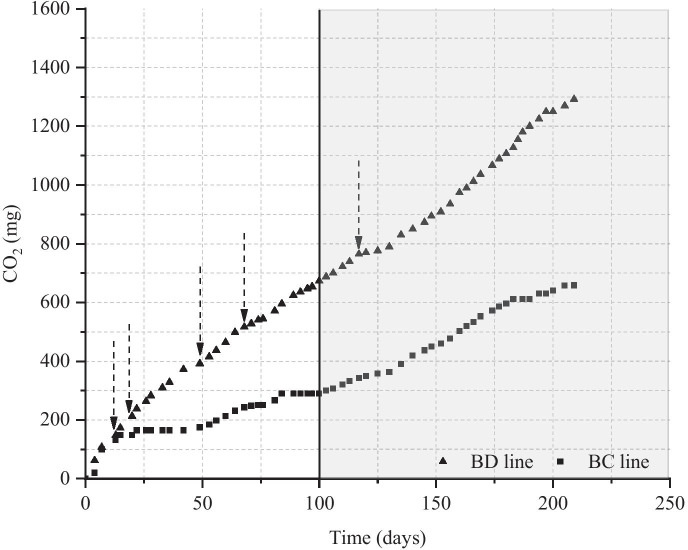
Total CO_2_ production trends in the BC and BD lines. Arrows indicate the CZ medium addition (without glucose and with a double salt concentration). The vertical line at 100th day indicates th*e supplementation of fresh soil*

Furthermore, whenever a slowdown in the CO_2_ production was observed, probably due to an unbalanced C/N ratio, 10 ml of CZ medium (without glucose and with a double concentration of salts) was added, in order to avoid a decrease in the microbial growth. In Fig. 4, the behaviour of the total CO_2_ production in the BC (squares in the graphic) and BD (triangles in the graphic) microcosms is reported. In either case, the addition of CZ medium had a positive effect on CO_2_ production; nevertheless, it was possible to observe that in the BD microcosms, the total CO_2_ produced was higher than in the BC ones. At the beginning of the incubation, about 13 days, the two curves were overlapped, probably in relation to the nutrients initially present in the soil and easily exploitable in both BC and BD microcosms. After the first addition of CZ medium (12 days), it was possible to observe a higher CO_2_ production in BD lines. The difference in CO_2_ production between BC and BD lines increased over incubation time and, after 100 days, the total CO_2_ produced in the BD microcosms was more than doubled (674 mg) than that of the biotic controls (290 mg). Very probably, this difference could be explained by the presence of NR as additional C-source in the BD microcosms.

#### Supplementation of Fresh Soil

After 99 days of incubation, the soil amount in the microcosms was almost halved due to sampling for humidity and pH monitoring, and to microbiological assays. At that time, it was brought back to the initial value (200 g) by means of the supplementation of fresh soil (the same as that used for microcosm set-up). The RH value was adjusted to 35% by adding distilled water. In all the microcosm types (i.e. BC and BD lines), the soil addition promoted the biomass growth (see Fig. 4). As it is possible to observe from the total CO_2_ production trend, the positive effect of fresh soil supplementation and that of the organic nutrient and salt addition increased the microbial activity in both microcosm type. This aspect was particularly evident in BC microcosms where a marked decrease in CO_2_ production was registered between the 80th and the 99th day of incubation: after the fresh soil addition (100th day), a recovery in the CO_2_ production was evident even though with a rate lower than that of the BD line.

#### Aeration of the Soil

**Fig. 5:**
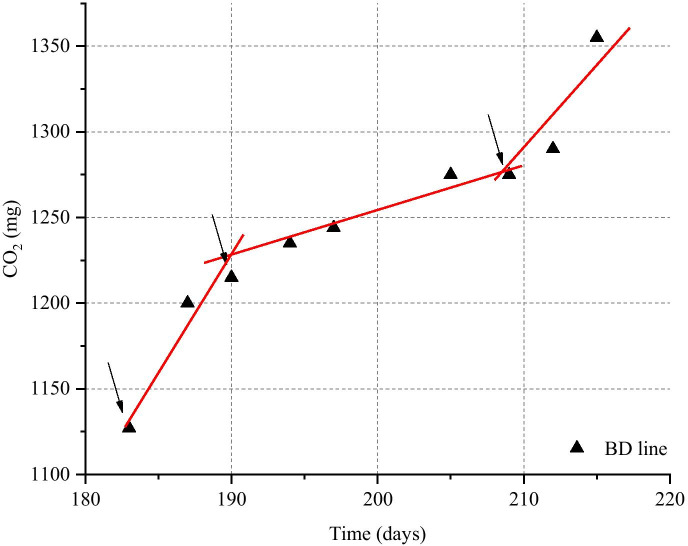
Influence of the soil aeration on the total CO_2_ production trend

During the incubation time, the silty loam soil induced the water stratification at the interface with the gas phase, hindering the O_2_ exchange between gas phase and soil. As a consequence, the aerobic metabolism, which is the main biodegradation mechanism, was inhibited. To avoid that, from the 183rd day of incubation, the soil was periodically mixed in each microcosm. As it is shown in Fig. 5, in the 6 days after the first mixing, the CO_2_ production rate grew quickly (14.7 mg_CO2_/day). After that, the microcosms were left, without any mixing, for a longer incubation period of 18 days, during which the CO_2_ production diminished to a rate of 3.3 mg_CO2_/day. In particular, between the 205th and 209th day, a steady state was registered. Nevertheless, after the third mixing, performed at the 209th day, the CO_2_ production rate had a resumption, and it reached a value of 13.3 mg_CO2_/day, very similar to that obtained after the first mixing.

#### Effect of NR Addition in BC Microcosms

In order to confirm the stimulating effect of NR on biodegrading indigenous microorganisms, after 68 days of incubation, the microcosms of the BC line were diversified and the effect of different C-source addition (i.e. glucose and NR) was evaluated. More precisely, the inorganic salts of the CZ medium were added in all the microcosms: BC one remained unvaried, glucose was added in BC-G microcosm, and NR sheets were positioned in the soil of the BC-NR microcosm.

**Fig. 6:**
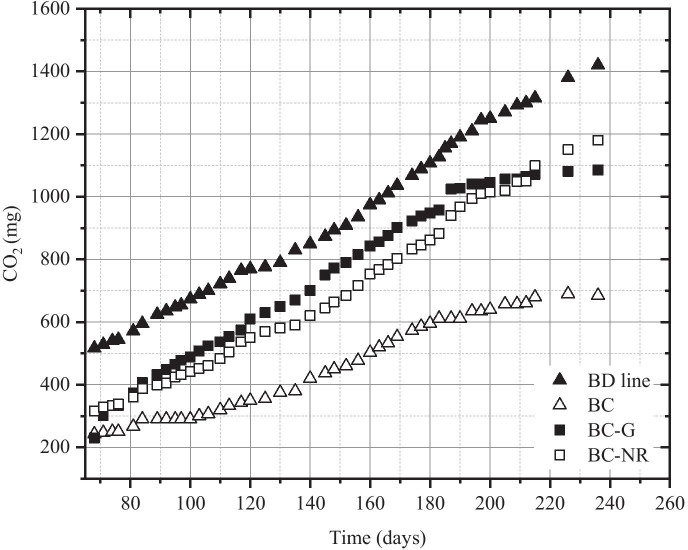
CO_2_ production trends in the BD line and in the BC lines added or not with C-source (glucose or NR)

During the incubation time, the microcosms showed a different behaviour (Fig. 6). At the 12th day of incubation, BC (white triangles) had the lowest CO_2_ production rate, 2 mg_CO2_/day, and BC-G (black squares) showed a foreseeable increase, 12 mg_CO2_/day, due to the addition of the easily usable C-source. On the contrary, BC-NR (white squares) showed a CO_2_ production rate, 3.75 mg_CO2_/day, similar to that of the BD line, 4.58 mgCO_2_/day (black triangles), confirming that the NR represents, for the soil microorganisms, a C-source that can be biodegraded but not easy to be metabolised. This fact was also confirmed by the trends of the respirometric curves in the last 36 days of incubation (200th ÷ 236th day): while the BC and BC-G curves showed a significant slowdown in CO_2_ production rate followed by a steady state, 1.25 mg_CO2_/day and 1.11 mg_CO2_/day, respectively, the BD and BC-NR curves were still growing with a comparable rate, 4.72 and 4.55 mg_CO2_/day respectively. These behaviours permit to describe the NR biodegradation as a slow but continuous phenomenon. Furthermore, the CO_2_ production rate of the BC-NR curve (4.55 mg_CO2_/day), in these last days of incubation, was very similar to that of the BD line (4.58 mg_CO2_/day) at the 12th day.

Although the respirometry trend of BC-NR was comparable to that of the BD microcosms, after 100 days of incubation, the total dry weight loss of NR sheets was 3.8%, about a half of that obtained, in the same period, in the microcosms of BD line (7.5%), as it will be described in the paragraph 3.4.2.

### Analysis on NR Sheets

The extent of the biodegradation of the NR sheets was evaluated by means of dry weight loss calculation; moreover, SEM micrographs of the samples, pre- and post- incubation, were performed in order to characterise the morphological changes of the rubber surface due to the biodegradation process.

#### Determination of NR Dry Weight Loss

The dry weight of all the NR samples was measured before the preparation of the microcosms. During the incubation period, at the time specified in Table 5, one NR sheet was extracted from each of the three BC and BD microcosm and washed to remove soil particles and biomass. The dry weight of each sheet was assessed again using the procedure described in the Materials and Methods section.

**Table 5:** Dry weight loss % of the NR sheets in the BC-NR microcosm *and BD line*

Time (days)	BC-NR microcosm	BD line
76	1.9 ± 0.3	7.5 ± 0.5
163	2.2 ± 0.3	12.3 ± 1.2
197	3.1 ± 0.1	12.9 ± 1.4
236	3.7 ± 0.1	15.6 ± 2.5

In Table 5, the results obtained in the BD line microcosms were compared with those of the BC-NR microcosm. From the reported values, it is possible to observe that the weight loss % of NR sheets, derived from BD microcosms, was always higher than those of the BC-NR ones; therefore, the weight loss caused by the biodegradation activity prevailed over the chemical degradation. At the end of the incubation period, 236 days, the weight loss in the BC-NR microcosm was equal to 3.7%, about one-quarter of that of the BD line, 15.6%. In the BD microcosm, NR sheets were added at the beginning of the test, thus explaining the greater weight loss, while in the BC-NR one, they were positioned 70 days later. Furthermore, as regard BD microcosm is concerned, the longer incubation time, in the presence of NR, has allowed the selection of the NR biodegrading biomass.

The result obtained in the BD line can be compared with that reported by Mastalygina et al. (2020), who described a NR degradation experiment by means of a full-scale soil test. They found a similar NR dry weight loss, 16.2%, in an incubation period of 45 days, that is five-fold lower than that reported in the present work. It has to be underlined that the soil, used in the mentioned work, was formulated with rich portions such as garden soil and horse manure. On the contrary, in the present work, a poor silty loam soil was used without any further modification. Regarding this aspect, different authors have reported that the biodegradation rate depends, among different factors, on the soil type (Doi & Fukuda, 2013; Grima et al., 2000).

The percentage values of weight loss, obtained at 163, 197, and 236 days of incubation, were probably underestimated because the washing method, suitable for NR sheets sampled at 76 days, has proved ineffective to remove soil particles and biomass from the surface of rubber incubated for longer times. In this last case, the filamentous biomass penetrated inside the NR structure embedding soil particles and making the washing difficult. On the contrary, on the NR sheets, picked up at 76 days of incubation, the biomass weakly adhered to the surface, and consequently, it was easier to be removed. These results, obtained at the two incubation times, were also evidenced by SEM analyses, as reported in the 3.4.2 paragraph.

#### SEM Micrographs

**Fig. 7:**
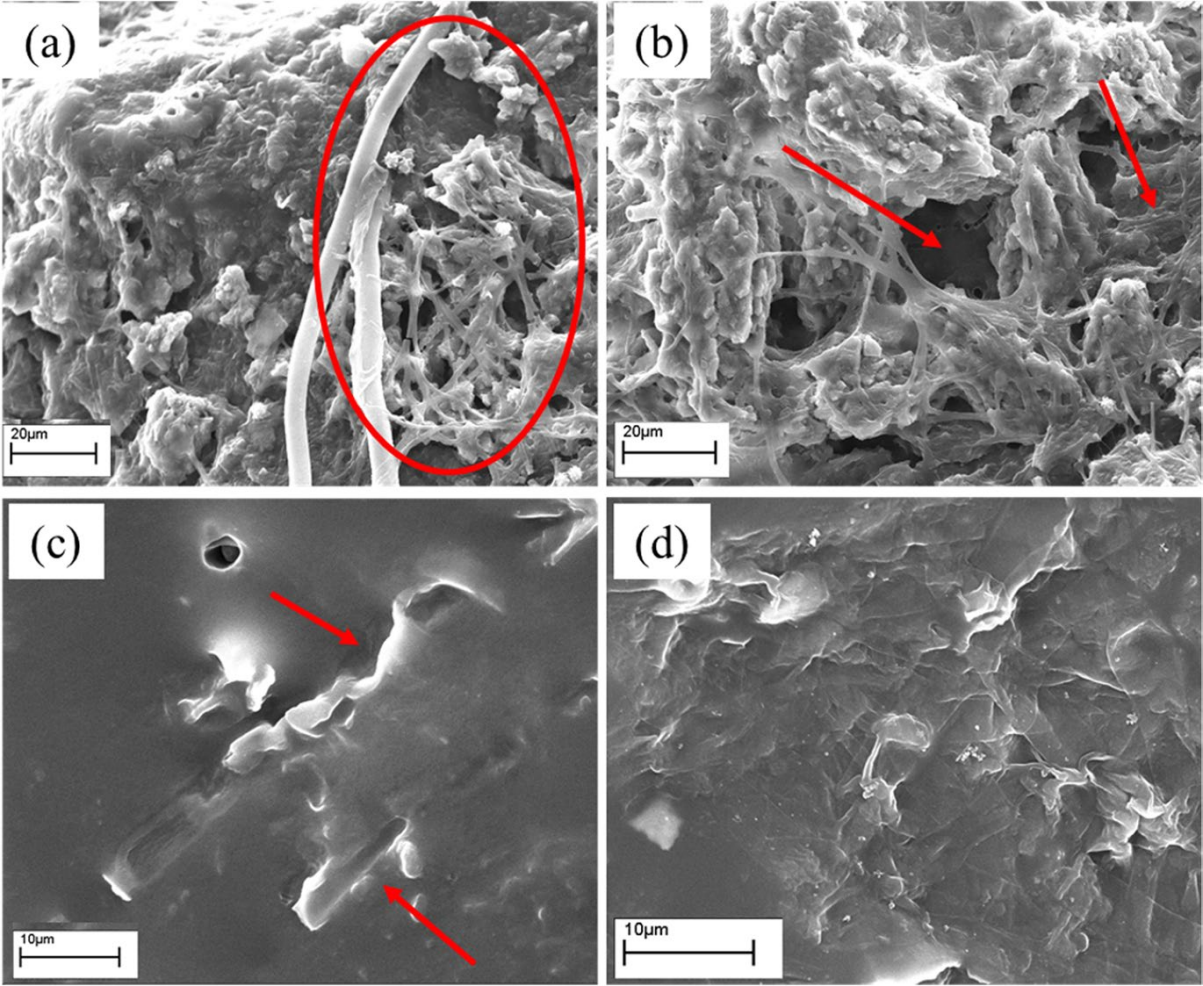
SEM micrographs of the developed mycelium on the NR surface (SE mode). ***a***, ***b*** Non-washed NR after 42 and 236 days of incubation respectively, while ***c***, ***d*** are the related washed samples

The extent of the microbial colonisation, in particular the fungal one, has been evaluated in the time course of the biodegradation experiment by means of SEM micrographs. NR sheets have been periodically sampled from the microcosms; sample fragments were cut and analysed without any treatment (fragments have only been gently cleaned to remove coarse soil particles) or after the washing procedure (see the Material and Methods section) to remove all the biomass which had colonised the rubber. As it is shown in Fig. 7, after 42 days of incubation, the mycelium was evident on limited areas of the rubber surface; moreover, the colonisation was mainly superficial (see the right side of the (a) micrograph). On the contrary, at the end of the incubation time, 236 days, the mycelium layer was thicker; it covered extensively all the rubber surface, and the diffused network of hyphae was strongly anchored to the surface (as shown in the left side of the (b) micrograph). The width of the fungal attachment to the NR surface has also been evidenced by the results of the washing procedure at the two incubation times. After 42 days, the rubber appears clean with outward footprints of the removed hyphae (as indicated by the arrows in Fig. 7c) showing the efficacy of the wash. On the contrary, after 236 days, the mycelium was strictly connected to the rubber and, for this reason, a layer of anchored hyphae remained strictly attached to the surface even after the vigorous washing treatment (Fig. 7d).

## Conclusions

In the present work, microcosm tests were prepared to verify the capability of the soil indigenous microflora to biodegrade natural rubber. To this purpose, NR sheets were incubated for about 6 months within soil microcosms, and biomass activity and growth had been evaluated by means of respirometric activity and microbial counts, while NR degradation was determined by means of dry weight loss and SEM analysis.

From respirometric measurements, it was established that the indigenous soil biomass was able to use the NR as the only C-source, since the CO_2_ production in the microcosms containing the NR samples was considerably higher than those without them. The dry weight loss of the incubated NR sheets confirmed that the “naturally selected” microbial consortium was able to efficiently biodegrade the polymer: after 236 days of incubation, the NR samples of the microcosms of the biodegradation line (BD) showed a dry weight loss of 15.6%, about four-fold higher than that obtained in the biotic control added with NR (3.7% in the BC-NR microcosm).

SEM micrographs highlighted the morphological changes occurred on the NR surface and allowed to observe that the fungal hyphae penetrated into the rubber by creating channels and folds; this phenomenon requires an incubation time quite long, in the order of about 2 months.

Biodegradation is mainly carried out by aerobic biomass, especially filamentous fungi; therefore, it was essential to periodically mix the soil to ensure a homogeneous distribution of O_2_ and the maintenance of the aerobic metabolism for long time periods. For the microorganisms, NR appears to be a source of C, immediately available even though difficult to be used, making the biodegradation a continuous but very slow phenomenon (order of months).

Since the specific surface is one of the main variables that control the yield of biodegradation in a granular solid phase, it is necessary to provide a pre-treatment of NR to reduce its size.

The results obtained in the microcosm study, carried out at laboratory scale, provided useful information in terms of soil aeration and nutrient amendment in view of a future scale-up of the biodegradation process.
